# Supplementary material for: A Transfected Babesia bovis Parasite Line Expressing eGFP Is Able to Complete the Full Life Cycle of the Parasite in Mammalian and Tick Hosts
Source: Pathogens. 2022 May 27;11(6):623. doi: 10.3390/pathogens11060623 (PMC9229605; doi:10.3390/pathogens11060623)
Supplement: Supplementary file 1 [file pathogens-11-00623-s001.zip › pathogens-1743168-supplementary.pdf]

AM sequence appended to 3' end of BBOV_II005480 base pair position

ATGTGCCAAGATCCTCAACGCAAAGGCAACGTGATACTCTCTCAGGCTTACGGGTGCCAAGATCCTCAACGCAAA
GGCAACGTGATACTCTCTCAGGCTTACTAG

PCR amplicons sequences

Seq 1. TfBbo5480 acquisition PCR 1

ACAAAATTTCAATTGCACGTTAAACCTGCTATTATAAGGATTGCCATATAATAGTGTACGTGAGTCAGTATCATATT
GTCAACAGGAAAACACAAATTATTTTAGAGAACCAATAGTATGAAAACATTTTCGACAACTGTGTAACGCTTTTTT
GCAATGCATTATGCGGTGCATAATGCCTGAGCTCGAATTAGCGACATGTAAAATACACCCAAAAACGTCTGAGAC
GTGGGTTTCATTATTGTACTAGACAGATTCAGAGTTAAACAACAGCTAAAGCTCATTAGATGCTTTGTTAAGTGATT
CTTCAAAATGTCTCTACGACATCTATAATTTAAAGATTGCGTCTTTGGTAGACACGGGTAATTATCAACGAATCAAC
AATTCATGGCCTTGCCGGTCTATTCAACATTCTAAATAGATGTATTCCAGTGGCCTGCTTTACAGGTTATCGCCAAG
ACAATTCTAATAACGAAGATAGTGGTGTCCATGGAATCTGCAACCCGTGCGGTGCAGATGGCGTACGCGTCTACA
TAGGTAGAAAGTGGTAATAGTGATTTTACCGGATACGATAGTATCTTCTAGGGTTACATTAGCTACATGACATCAC
AATCATTCACGTCTTAAATTCAGTAAATCTTGGGTCTCGCGTTACCACAGCTCGTCTATTGGGCAATAATATTTAGA
CCCCCACCATCGGGGGCGCTCCGTCGCAGGTAGTAAATATTCACTCGAAGGGAAATATAGCGCATCCAAAGTGGC
GTTATATTTCTATCAACACGATCAAAACTGCTTTAAGGAGGGCAAAGCACCCTTCTTTAAAATTTTAACTTACTTATT
TACCTTTATTTAGCCCAAAGAGGGATAAGGTCTTATTGGCTGTACGAGGGCCCATGTCACTGACTGCTCAGCCCGA
ACACGTTGCACAGGAAGTGCCTACTCACGTTATTCCCTATTCTGAGAACTACATGGGATATTACCCTGGTGATATG
AGTGCAGGTCTACAATGTCACGGTTACCTGCCTTCTCCTCAATACACTCAATATTATGATGACCCTTACATGGGTAC
AGCGGTTGACTACATGAACGTCACCTGTTACCAGATGGTCCCAGATGACCATCATGGTTACGTCGACTCTCATGTT
GGACAAATGCCCGACTACTCCGGTGGCATGATGTACCATCCTATGGAGATGACATACTGCCCTCCTACTGGGCAGA
TGTACTATCAAACACCTGGATCGAGTACCGTACCGGCATTAAGCAGCGTCAAGGGGGATCCCGATGACGATGCTC
GTACTTTTACTGAATGCAGTACTGCGGCAACTACTCCTAACTTGGAACACTGCCCCAACCCCGAAGTCGTTGAAGA
TGGATTTGATCTTGGTTATGGATACGACTACGACCGATACGCAGTATCACCTTCTGACTGCTACCCTCGCCAAAGTG
CTGGGTTTAGCGGTTACCCTGGTTACTGTGTGTCCAACTTTGACGATGTATTTTCCGCTTGTGGCGACCAGGACCCT
TTAGTGAACGATGCTATGGTTGTGGAGATTGCTGACGAGATAGCGGCCAACGTCCAGTATTTACCAGACAAGGAA
TCCAGTGGTAAATACTCACTTGACAAAAACCACCCGATTCATTGCGTCTGGCGTGATCTCAACAGAGGTCACTGCA
GTTGGCGTTGCCGCTGGTGGGAGAACGGCAAGCGTTTGAGTAAGAACTTCAACGTGAAACGCTTCGGTGAGCAT
GAGGCCATGTGGATGGCCATTGCTATGAAGATTCGTAACTCTACGCCTGTTGAGCGTTTTCAGTTACTCAAGGAGC
AGCGTGAAGCTGTTCGCAACTACTGTGAGCTTGTGCGTCAAAACGTTGATGGGTCGGTTGTTGACTATCGTGTTGA
TCCAAAAAGTTCTATTGCTATAGCTCAGATTGGCACTGCTACGCGCAAGGCCCGATCACCTCTGAACAGCACTAAG
AGTGCTCAGAAGGCCAACTGGGCGCGCATCGCTTGGACCTATGTTATGGTTTCTGAACGTCGTTCTGCTGCTAGCA
GTGTAGTTTGTCGTCTATGTGCGAAGACTACTAAATGCTCTCGCAGTCGTAACCTGCAACAGGCGCATATAATGCA
CCTCATTCGTATGCACAAGAACCCTTGGAAGCAGTATTTGGATGAGGAGGGACTCACTAAAATGTGTAGATCCGCT
CTGGATGCTTTGTATCAGCAGCGTGGTTTTACTGCTACAAGTGAATGGAAGTTGAGGCCGGGTCTCACTCCACCTG
AGCCTGGGTTCGATTGTTCTTCAATGGTTGATGGTGATAGTGTTTTGGATGCTAGCATGTGCCAAGATCCTCAACG
CAAAGGCAACGTGATACTCTCTCAGGCTTACGGGTGCCAAGATCCTCAACGCAAAGGCAACGTGATACTCTCTCAG
GCTTACTAGCCGCGGTCTAGAGGATCCTAATGGCACAAACTCAACAAATGATGTATCGTCATCTGATCCATCGGTT
TTCAATATTGTATTGGATGCAATATCTGAATGCATATGATGCGACAGTTTCCATCATCGGGTGCCGAATCGTAACTC
TCATAACACCATTTTAAGTTATGTAAACTAGTATCTATGTTTATGGTTTACGTAAAATGAATGTTTGAGTCTACAGG

AAGGAACACGTGTGTAGAAGTATTTATTTCGTGAATGTACGTCATATATTTTTATGTATACGTGAACCTCACTAAAG
GAAATTTTTGATGTTTTACGTATGCATTTATTAAACAACACTCTGTTAAATTATTCCATACGCTAAGATATGATTCCT
TTCTCGCAAATCTCCCCGGGCTGCTGGAAAAAAATCAATTGATATATAAATAAACTTATATAAACCACGTAATAAAT
GAGATAAATAAATATATGTCATGTATAAATTTGGGGTATAATTTTAATTGTAGAAGGTGGAGATAGTACGGTAAAA
ACTGAGATGATATATAATATATGATGAATATATAAACTTGACAGGTCAAGATTACTTGATCAGATTTAATTAGTTGT
GATAAAGCGGAAATTCCCATATATGTCATCCCGTTAAAGGTATTAATAGCATTTTAAGAATATATTTCACAAATATG
ATATACCATACATCTGATAGAATTATGCATCGATATTCGTCGTTATAGGCTTAGTTGCGCACAACGAAAAGGTATG
TGTATATACTTAATTATATTTCAATCAAACGATGGGTGACAAATGTGTGGCGTGGAGCAAAGCAAAACAGCACCAC
AACCTTTACAAAGGGATCTTCATTAGTCCTTTGCAGTGTCTTTATAACTTAATAAAGTAATTCCACGCAAGAATAGT
GATTATATCGCCAACAACGCACTTTTGTGATGTAAGTCAGGCCCCTGTCATTTTGCTTTGTGGGTGGTGTTATTTCA
TGAAGCAGGCGACTACTTCTTGTCGAGCTGCTGTGTGTATATTCTACTTTTGAGGCTATTATATACACTGATGTGTT
TAGACATTTGTGTTATTTAATGCTTGATTTAACGTTTTTTCAATTGTGTTTGCCGATACTAATGTTGTCTCAGATATA
ATTTATTGCAACTTTACGAAAATAAGCTTATGGTGAGCAAGGGCGAGGAGCTGTTCACCGGGGTGGTGCCCATCC
TGGTCGAGCTGGACGGCGACGTAAACGGCCACAAGTTCAGCGTGTCCGGCGAGGGCGAGGGCGATGCCACCTAC
GGCAAGCTGACCCTGAAGTTCATCTGCACCACCGGCAAGCTGCCCGTGCCCTGGCCCACCCTCGTGACCACCCTGA
CCTACGGCGTGCAGTGCTTCAGCCGCTACCCCGACCACATGAAGCAGCACGACTTCTTCAAGTCCGCCATGCCCGA
AGGCTACGTCCAGGAGCGCACCATCTTCTTCAAGGACGACGGCAACTACAAGACCCGCGCCGAGGTGAAGTT

Seq 2. TfBbo5480 transmission PCR 1

ACAAAATTTCAATTGCACGTTAAACCTGCTATTATAAGGATTGCCATATAATAGTGTACGTGAGTCAGTATCATATT
GTCAACAGGAAAACACAAATTATTTTAGAGAACCAATAGTATGAAAACATTTTCGACAACTGTGTAACGCTTTTTT
GCAATGCATTATGCGGTGCATAATGCCTGAGCTCGAATTAGCGACATGTAAAATACACCCAAAAACGTCTGAGAC
GTGGGTTTCATTATTGTACTAGACAGATTCAGAGTTAAACAACAGCTAAAGCTCATTAGATGCTTTGTTAAGTGATT
CTTCAAAATGTCTCTACGACATCTATAATTTAAAGATTGCGTCTTTGGTAGACACGGGTAATTATCAACGAATCAAC
AATTCATGGCCTTGCCGGTCTATTCAACATTCTAAATAGATGTATTCCAGTGGCCTGCTTTACAGGTTATCGCCAAG
ACAATTCTAATAACGAAGATAGTGGTGTCCATGGAATCTGCAACCCGTGCGGTGCAGATGGCGTACGCGTCTACA
TAGGTAGAAAGTGGTAATAGTGATTTTACCGGATACGATAGTATCTTCTAGGGTTACATTAGCTACATGACATCAC
AATCATTCACGTCTTAAATTCAGTAAATCTTGGGTCTCGCGTTACCACAGCTCGTCTATTGGGCAATAATATTTAGA
CCCCCACCATCGGGGGCGCTCCGTCGCAGGTAGTAAATATTCACTCGAAGGGAAATATAGCGCATCCAAAGTGGC
GTTATATTTCTATCAACACGATCAAAACTGCTTTAAGGAGGGCAAAGCACCCTTCTTTAAAATTTTAACTTACTTATT
TACCTTTATTTAGCCCAAAGAGGGATAAGGTCTTATTGGCTGTACGAGGGCCCATGTCACTGACTGCTCAGCCCGA
ACACGTTGCACAGGAAGTGCCTACTCACGTTATTCCCTATTCTGAGAACTACATGGGATATTACCCTGGTGATATG
AGTGCAGGTCTACAATGTCACGGTTACCTGCCTTCTCCTCAATACACTCAATATTATGATGACCCTTACATGGGTAC
AGCGGTTGACTACATGAACGTCACCTGTTACCAGATGGTCCCAGATGACCATCATGGTTACGTCGACTCTCATGTT
GGACAAATGCCCGACTACTCCGGTGGCATGATGTACCATCCTATGGAGATGACATACTGCCCTCCTACTGGGCAGA
TGTACTATCAAACACCTGGATCGAGTACCGTACCGGCATTAAGCAGCGTCAAGGGGGATCCCGATGACGATGCTC
GTACTTTTACTGAATGCAGTACTGCGGCAACTACTCCTAACTTGGAACACTGCCCCAACCCCGAAGTCGTTGAAGA
TGGATTTGATCTTGGTTATGGATACGACTACGACCGATACGCAGTATCACCTTCTGACTGCTACCCTCGCCAAAGTG
CTGGGTTTAGCGGTTACCCTGGTTACTGTGTGTCCAACTTTGACGATGTATTTTCCGCTTGTGGCGACCAGGACCCT
TTAGTGAACGATGCTATGGTTGTGGAGATTGCTGACGAGATAGCGGCCAACGTCCAGTATTTACCAGACAAGGAA
TCCAGTGGTAAATACTCACTTGACAAAAACCACCCGATTCATTGCGTCTGGCGTGATCTCAACAGAGGTCACTGCA
GTTGGCGTTGCCGCTGGTGGGAGAACGGCAAGCGTTTGAGTAAGAACTTCAACGTGAAACGCTTCGGTGAGCAT
GAGGCCATGTGGATGGCCATTGCTATGAAGATTCGTAACTCTACGCCTGTTGAGCGTTTTCAGTTACTCAAGGAGC
AGCGTGAAGCTGTTCGCAACTACTGTGAGCTTGTGCGTCAAAACGTTGATGGGTCGGTTGTTGACTATCGTGTTGA

TCCAAAAAGTTCTATTGCTATAGCTCAGATTGGCACTGCTACGCGCAAGGCCCGATCACCTCTGAACAGCACTAAG
AGTGCTCAGAAGGCCAACTGGGCGCGCATCGCTTGGACCTATGTTATGGTTTCTGAACGTCGTTCTGCTGCTAGCA
GTGTAGTTTGTCGTCTATGTGCGAAGACTACTAAATGCTCTCGCAGTCGTAACCTGCAACAGGCGCATATAATGCA
CCTCATTCGTATGCACAAGAACCCTTGGAAGCAGTATTTGGATGAGGAGGGACTCACTAAAATGTGTAGATCCGCT
CTGGATGCTTTGTATCAGCAGCGTGGTTTTACTGCTACAAGTGAATGGAAGTTGAGGCCGGGTCTCACTCCACCTG
AGCCTGGGTTCGATTGTTCTTCAATGGTTGATGGTGATAGTGTTTTGGATGCTAGCATGTGCCAAGATCCTCAACG
CAAAGGCAACGTGATACTCTCTCAGGCTTACGGGTGCCAAGATCCTCAACGCAAAGGCAACGTGATACTCTCTCAG
GCTTACTAGCCGCGGTCTAGAGGATCCTAATGGCACAAACTCAACAAATGATGTATCGTCATCTGATCCATCGGTT
TTCAATATTGTATTGGATGCAATATCTGAATGCATATGATGCGACAGTTTCCATCATCGGGTGCCGAATCGTAACTC
TCATAACACCATTTTAAGTTATGTAAACTAGTATCTATGTTTATGGTTTACGTAAAATGAATGTTTGAGTCTACAGG
AAGGAACACGTGTGTAGAAGTATTTATTTCGTGAATGTACGTCATATATTTTTATGTATACGTGAACCTCACTAAAG
GAAATTTTTGATGTTTTACGTATGCATTTATTAAACAACACTCTGTTAAATTATTCCATACGCTAAGATATGATTCCT
TTCTCGCAAATCTCCCCGGGCTGCTGGAAAAAAATCAATTGATATATAAATAAACTTATATAAACCACGTAATAAAT
GAGATAAATAAATATATGTCATGTATAAATTTGGGGTATAATTTTAATTGTAGAAGGTGGAGATAGTACGGTAAAA
ACTGAGATGATATATAATATATGATGAATATATAAACTTGACAGGTCAAGATTACTTGATCAGATTTAATTAGTTGT
GATAAAGCGGAAATTCCCATATATGTCATCCCGTTAAAGGTATTAATAGCATTTTAAGAATATATTTCACAAATATG
ATATACCATACATCTGATAGAATTATGCATCGATATTCGTCGTTATAGGCTTAGTTGCGCACAACGAAAAGGTATG
TGTATATACTTAATTATATTTCAATCAAACGATGGGTGACAAATGTGTGGCGTGGAGCAAAGCAAAACAGCACCAC
AACCTTTACAAAGGGATCTTCATTAGTCCTTTGCAGTGTCTTTATAACTTAATAAAGTAATTCCACGCAAGAATAGT
GATTATATCGCCAACAACGCACTTTTGTGATGTAAGTCAGGCCCCTGTCATTTTGCTTTGTGGGTGGTGTTATTTCA
TGAAGCAGGCGACTACTTCTTGTCGAGCTGCTGTGTGTATATTCTACTTTTGAGGCTATTATATACACTGATGTGTT
TAGACATTTGTGTTATTTAATGCTTGATTTAACGTTTTTTCAATTGTGTTTGCCGATACTAATGTTGTCTCAGATATA
ATTTATTGCAACTTTACGAAAATAAGCTTATGGTGAGCAAGGGCGAGGAGCTGTTCACCGGGGTGGTGCCCATCC
TGGTCGAGCTGGACGGCGACGTAAACGGCCACAAGTTCAGCGTGTCCGGCGAGGGCGAGGGCGATGCCACCTAC
GGCAAGCTGACCCTGAAGTTCATCTGCACCACCGGCAAGCTGCCCGTGCCCTGGCCCACCCTCGTGACCACCCTGA
CCTACGGCGTGCAGTGCTTCAGCCGCTACCCCGACCACATGAAGCAGCACGACTTCTTCAAGTCCGCCATGCCCGA
AGGCTACGTCCAGGAGCGCACCATCTTCTTCAAGGACGACGGCAACTACAAGACCCGCGCCGAGGTGAAGTT

Seq 3. **TfBbo5480 acquisition PCR 2**

CATATATGTCATCCCGTTAAAGGTATTAATAGCATTTTAAGAATATATTTCACAAATATGATATACCATACATCTGAT
AGAATTATGCATCGATATTCGTCGTTATAGGCTTAGTTGCGCACAACGAAAAGGTATGTGTATATACTTAATTATAT
TTCAATCAAACGATGGGTGACAAATGTGTGGCGTGGAGCAAAGCAAAACAGCACCACAACCTTTACAAAGGGATC
TTCATTAGTCCTTTGCAGTGTCTTTATAACTTAATAAAGTAATTCCACGCAAGAATAGTGATTATATCGCCAACAAC
GCACTTTTGTGATGTAAGTCAGGCCCCTGTCATTTTGCTTTGTGGGTGGTGTTATTTCATGAAGCAGGCGACTACTT
CTTGTCGAGCTGCTGTGTGTATATTCTACTTTTGAGGCTATTATATACACTGATGTGTTTAGACATTTGTGTTATTTA
ATGCTTGATTTAACGTTTTTTCAATTGTGTTTGCCGATACTAATGTTGTCTCAGATATAATTTATTGCAACTTTACGA
AAATAAGCTTATGGTGAGCAAGGGCGAGGAGCTGTTCACCGGGGTGGTGCCCATCCTGGTCGAGCTGGACGGCG
ACGTAAACGGCCACAAGTTCAGCGTGTCCGGCGAGGGCGAGGGCGATGCCACCTACGGCAAGCTGACCCTGAAG
TTCATCTGCACCACCGGCAAGCTGCCCGTGCCCTGGCCCACCCTCGTGACCACCCTGACCTACGGCGTGCAGTGCT
TCAGCCGCTACCCCGACCACATGAAGCAGCACGACTTCTTCAAGTCCGCCATGCCCGAAGGCTACGTCCAGGAGC
GCACCATCTTCTTCAAGGACGACGGCAACTACAAGACCCGCGCCGAGGTGAAGTTCGAGGGCGACACCCTGGTGA
ACCGCATCGAGCTGAAGGGCATCGACTTCAAGGAGGACGGCAACATCCTGGGGCACAAGCTGGAGTACAACTAC
AACAGCCACAACGTCTATATCATGGCCGACAAGCAGAAGAACGGCATCAAGGTGAACTTCAAGATCCGCCACAAC
ATCGAGGACGGCAGCGTGCAGCTCGCCGACCACTACCAGCAGAACACCCCCATCGGCGACGGCCCCGTGCTGCTG

CCCGACAACCACTACCTGAGCACCCAGTCCGCCCTGAGCAAAGACCCCAACGAGAAGCGCGATCACATGGTCCTG
CTG

Seq 4. TfBbo5480 transmission PCR 2

CATATATGTCATCCCGTTAAAGGTATTAATAGCATTTTAAGAATATATTTCACAAATATGATATACCATACATCTGAT
AGAATTATGCATCGATATTCGTCGTTATAGGCTTAGTTGCGCACAACGAAAAGGTATGTGTATATACTTAATTATAT
TTCAATCAAACGATGGGTGACAAATGTGTGGCGTGGAGCAAAGCAAAACAGCACCACAACCTTTACAAAGGGATC
TTCATTAGTCCTTTGCAGTGTCTTTATAACTTAATAAAGTAATTCCACGCAAGAATAGTGATTATATCGCCAACAAC
GCACTTTTGTGATGTAAGTCAGGCCCCTGTCATTTTGCTTTGTGGGTGGTGTTATTTCATGAAGCAGGCGACTACTT
CTTGTCGAGCTGCTGTGTGTATATTCTACTTTTGAGGCTATTATATACACTGATGTGTTTAGACATTTGTGTTATTTA
ATGCTTGATTTAACGTTTTTTCAATTGTGTTTGCCGATACTAATGTTGTCTCAGATATAATTTATTGCAACTTTACGA
AAATAAGCTTATGGTGAGCAAGGGCGAGGAGCTGTTCACCGGGGTGGTGCCCATCCTGGTCGAGCTGGACGGCG
ACGTAAACGGCCACAAGTTCAGCGTGTCCGGCGAGGGCGAGGGCGATGCCACCTACGGCAAGCTGACCCTGAAG
TTCATCTGCACCACCGGCAAGCTGCCCGTGCCCTGGCCCACCCTCGTGACCACCCTGACCTACGGCGTGCAGTGCT
TCAGCCGCTACCCCGACCACATGAAGCAGCACGACTTCTTCAAGTCCGCCATGCCCGAAGGCTACGTCCAGGAGC
GCACCATCTTCTTCAAGGACGACGGCAACTACAAGACCCGCGCCGAGGTGAAGTTCGAGGGCGACACCCTGGTGA
ACCGCATCGAGCTGAAGGGCATCGACTTCAAGGAGGACGGCAACATCCTGGGGCACAAGCTGGAGTACAACTAC
AACAGCCACAACGTCTATATCATGGCCGACAAGCAGAAGAACGGCATCAAGGTGAACTTCAAGATCCGCCACAAC
ATCGAGGACGGCAGCGTGCAGCTCGCCGACCACTACCAGCAGAACACCCCCATCGGCGACGGCCCCGTGCTGCTG
CCCGACAACCACTACCTGAGCACCCAGTCCGCCCTGAGCAAAGACCCCAACGAGAAGCGCGATCACATGGTCCTG
CTG

Seq 5. TfBbo5480 acquisition PCR 3

GAGCAACGGCTACAATCAACAGCATCCCCATCTCTGAAGACTACAGCGTCGCCAGCGCAGCTCTCTCTAGCGACGG
CCGCATCTTCACTGGTGTCAATGTATATCATTTTACTGGGGGACCTTGTGCAGAACTCGTGGTGCTGGGCACTGCT
GCTGCTGCGGCAGCTGGCAACCTGACTTGTATCGTCGCGATCGGAAATGAGAACAGGGGCATCTTGAGCCCCTGC
GGACGGTGCCGACAGGTGCTTCTCGATCTGCATCCTGGGATCAAAGCCATAGTGAAGGACAGTGATGGACAGCC
GACGGCAGTTGGGATTCGTGAATTGCTGCCCTCTGGTTATGTGTGGGAGGGCTAAGAATTCATGATCGCTGATGG
TGCATAAAATAGTGTACCACGTTGTTTATTTTACTCTTTATATAAGGATAACATTCTTTGTTCTTCATCGATAATGTG
GTATAATACTGACTGCCGGTTTTATTGGTAATCATTTCGGCTCAGTTGCTTTTAGTACATCAATTTTAATTATTATCT
GTTTTATTTCGTTTTTATAGTGTTTTAGTTTCTAGCACTTATCAAATCACGGGGTTCGGTCGCATTACACTGCTATGT
GCTTTGAAATCGGTGTTTCGTTAGGCCTAATAATTACTAGTTACTATTATATGCAACGATTAACAGATAATTATATC
GGCAGTGGTTTGTTAAGCTTCTTGGCACGCTTTATTGCTCTGCGCCGAGCTTCCCATTCTTGATCGTGTGCTGCAGT
CTCCTTTAACTGTGCGTTCAGTCGTTCCATATATGCCTTCTTCTCTGTTGATATATCTACTAGTGTAGGTAGCTCATCT
TGATATCGCTCATCCTCAACTTCTTCATCTGAGTCTGCCTCAAACACCACATGCTTCTGTTCAGTTTGTTTGATTGTTT
CCACACCACGTAGTTTAGCAACACCATGCCTATTTAATCGGAATTTCTTCCTTGTTGATGCTAGGTTGGCTTTCAGCT
TTACATCTTCGTTATCCTCTGTTGCCTTTTGTTCTATGTAATGTTTCAGGTCGTCATCCACTCTCGAGGCATCAGTTCC
AACACGTGTGAGTATATTATCCGTGTCAGCGTCAGATTCATCGGACGATTCATCCGAATCCACGACAGTACTACTG
ACATCAGACTCACTTGCGATGTCCACCTTTGTTTCATTAGACAGCTGTTTCTTGGCTTTTATCTTTTCCTTTAGCTTCG
AGAGCTTCGAAGGCTTTTCGCTAGTATTGTCATTTACAGTTATTTGGGGTGCTGATGGCAATCCGCTGGCTAATGC
CATTTGCTGCACAGATTCAACGAGTTCAGCACCGGAGAGTGTAACAGATTTACGCGTGGATAGATAGCGCATATA

TGATATAACAGCGCGTTGTGCGATTTCCTTAATCCAGGCTTCCTTTGCCATTAAGGCCTGTAGTTTTTGTAGGACAT
AGTTTTCCTTGCGTTGTAGGAACGGCAATAGCAATTTCGTTAGGTTGTGCAACTTCACTCCAGATGTTTTGAGTTGT
TCCACGAATGATGCTTCATTTTCACTAATTATAGAGAATGCGATACCAAAGCTGCGCGTTCCTTCCACGGTCAACCT
ACCGGTACGGCCCACGCGATGCGTATATGTGTTGACAGAGTCTGGTATATCCAGCTGTACTACGTAATCCACTGCT
GGGAAGTCAACTCCACGTGATCCTACATCAGTAGTAAAAATACATCCATTATTTGCTTTGCTGCAAATCGAGTGA
ACTGTTCATTTCGTTTAAGTTGACTTTGTTTACCATGCCACTCCGTCATTGGCACTGCAGGGATCAGTCTTTTGAATA
CCTCGAAAACCAGTCTAACGTGCTTACACGTTGCTAGGAATACTATAAAGCGTTTGTTTTGATTCTTGCT

Seq 6. TfBbo5480 transmission PCR 3

GAGCAACGGCTACAATCAACAGCATCCCCATCTCTGAAGACTACAGCGTCGCCAGCGCAGCTCTCTCTAGCGACGG
CCGCATCTTCACTGGTGTCAATGTATATCATTTTACTGGGGGACCTTGTGCAGAACTCGTGGTGCTGGGCACTGCT
GCTGCTGCGGCAGCTGGCAACCTGACTTGTATCGTCGCGATCGGAAATGAGAACAGGGGCATCTTGAGCCCCTGC
GGACGGTGCCGACAGGTGCTTCTCGATCTGCATCCTGGGATCAAAGCCATAGTGAAGGACAGTGATGGACAGCC
GACGGCAGTTGGGATTCGTGAATTGCTGCCCTCTGGTTATGTGTGGGAGGGCTAAGAATTCATGATCGCTGATGG
TGCATAAAATAGTGTACCACGTTGTTTATTTTACTCTTTATATAAGGATAACATTCTTTGTTCTTCATCGATAATGTG
GTATAATACTGACTGCCGGTTTTATTGGTAATCATTTCGGCTCAGTTGCTTTTAGTACATCAATTTTAATTATTATCT
GTTTTATTTCGTTTTTATAGTGTTTTAGTTTCTAGCACTTATCAAATCACGGGGTTCGGTCGCATTACACTGCTATGT
GCTTTGAAATCGGTGTTTCGTTAGGCCTAATAATTACTAGTTACTATTATATGCAACGATTAACAGATAATTATATC
GGCAGTGGTTTGTTAAGCTTCTTGGCACGCTTTATTGCTCTGCGCCGAGCTTCCCATTCTTGATCGTGTGCTGCAGT
CTCCTTTAACTGTGCGTTCAGTCGTTCCATATATGCCTTCTTCTCTGTTGATATATCTACTAGTGTAGGTAGCTCATCT
TGATATCGCTCATCCTCAACTTCTTCATCTGAGTCTGCCTCAAACACCACATGCTTCTGTTCAGTTTGTTTGATTGTTT
CCACACCACGTAGTTTAGCAACACCATGCCTATTTAATCGGAATTTCTTCCTTGTTGATGCTAGGTTGGCTTTCAGCT
TTACATCTTCGTTATCCTCTGTTGCCTTTTGTTCTATGTAATGTTTCAGGTCGTCATCCACTCTCGAGGCATCAGTTCC
AACACGTGTGAGTATATTATCCGTGTCAGCGTCAGATTCATCGGACGATTCATCCGAATCCACGACAGTACTACTG
ACATCAGACTCACTTGCGATGTCCACCTTTGTTTCATTAGACAGCTGTTTCTTGGCTTTTATCTTTTCCTTTAGCTTCG
AGAGCTTCGAAGGCTTTTCGCTAGTATTGTCATTTACAGTTATTTGGGGTGCTGATGGCAATCCGCTGGCTAATGC
CATTTGCTGCACAGATTCAACGAGTTCAGCACCGGAGAGTGTAACAGATTTACGCGTGGATAGATAGCGCATATA
TGATATAACAGCGCGTTGTGCGATTTCCTTAATCCAGGCTTCCTTTGCCATTAAGGCCTGTAGTTTTTGTAGGACAT
AGTTTTCCTTGCGTTGTAGGAACGGCAATAGCAATTTCGTTAGGTTGTGCAACTTCACTCCAGATGTTTTGAGTTGT
TCCACGAATGATGCTTCATTTTCACTAATTATAGAGAATGCGATACCAAAGCTGCGCGTTCCTTCCACGGTCAACCT
ACCGGTACGGCCCACGCGATGCGTATATGTGTTGACAGAGTCTGGTATATCCAGCTGTACTACGTAATCCACTGCT
GGGAAGTCAACTCCACGTGATCCTACATCAGTAGTAAAAATACATCCATTATTTGCTTTGCTGCAAATCGAGTGA
ACTGTTCATTTCGTTTAAGTTGACTTTGTTTACCATGCCACTCCGTCATTGGCACTGCAGGGATCAGTCTTTTGAATA
CCTCGAAAACCAGTCTAACGTGCTTACACGTTGCTAGGAATACTATAAAGCGTTTGTTTTGATTCTTGCT

Seq 7: Figure 7 A- TF acquisition (3656 bp band)

ATCGCTTGGACCTATGTTATGGTTTCTGAACGTCGTTCTGCTGCTAGCAGTGTAGTTTGTCGTCTATGTGCGAAGAC
TACTAAATGCTCTCGCAGTCGTAACCTGCAACAGGCGCATATAATGCACCTCATTCGTATGCACAAGAACCCTTGG
AAGCAGTATTTGGATGAGGAGGGACTCACTAAAATGTGTAGATCCGCTCTGGATGCTTTGTATCAGCAGCGTGGT
TTTACTGCTACAAGTGAATGGAAGTTGAGGCCGGGTCTCACTCCACCTGAGCCTGGGTTCGATTGTTCTTCAATGG
TTGATGGTGATAGTGTTTTGGATGCTAGCATGTGCCAAGATCCTCAACGCAAAGGCAACGTGATACTCTCTCAGGC
TTACGGGTGCCAAGATCCTCAACGCAAAGGCAACGTGATACTCTCTCAGGCTTACTAGCCGCGGTCTAGAGGATCC

TAATGGCACAAACTCAACAAATGATGTATCGTCATCTGATCCATCGGTTTTCAATATTGTATTGGATGCAATATCTG
AATGCATATGATGCGACAGTTTCCATCATCGGGTGCCGAATCGTAACTCTCATAACACCATTTTAAGTTATGTAAAC
TAGTATCTATGTTTATGGTTTACGTAAAATGAATGTTTGAGTCTACAGGAAGGAACACGTGTGTAGAAGTATTTAT
TTCGTGAATGTACGTCATATATTTTTATGTATACGTGAACCTCACTAAAGGAAATTTTTGATGTTTTACGTATGCATT
TATTAAACAACACTCTGTTAAATTATTCCATACGCTAAGATATGATTCCTTTCTCGCAAATCTCCCCGGGCTGCTGGA
AAAAAATCAATTGATATATAAATAAACTTATATAAACCACGTAATAAATGAGATAAATAAATATATGTCATGTATAA
ATTTGGGGTATAATTTTAATTGTAGAAGGTGGAGATAGTACGGTAAAAACTGAGATGATATATAATATATGATGA
ATATATAAACTTGACAGGTCAAGATTACTTGATCAGATTTAATTAGTTGTGATAAAGCGGAAATTCCCATATATGTC
ATCCCGTTAAAGGTATTAATAGCATTTTAAGAATATATTTCACAAATATGATATACCATACATCTGATAGAATTATG
CATCGATATTCGTCGTTATAGGCTTAGTTGCGCACAACGAAAAGGTATGTGTATATACTTAATTATATTTCAATCAA
ACGATGGGTGACAAATGTGTGGCGTGGAGCAAAGCAAAACAGCACCACAACCTTTACAAAGGGATCTTCATTAGT
CCTTTGCAGTGTCTTTATAACTTAATAAAGTAATTCCACGCAAGAATAGTGATTATATCGCCAACAACGCACTTTTG
TGATGTAAGTCAGGCCCCTGTCATTTTGCTTTGTGGGTGGTGTTATTTCATGAAGCAGGCGACTACTTCTTGTCGAG
CTGCTGTGTGTATATTCTACTTTTGAGGCTATTATATACACTGATGTGTTTAGACATTTGTGTTATTTAATGCTTGAT
TTAACGTTTTTTCAATTGTGTTTGCCGATACTAATGTTGTCTCAGATATAATTTATTGCAACTTTACGAAAATAAGCT
TATGGTGAGCAAGGGCGAGGAGCTGTTCACCGGGGTGGTGCCCATCCTGGTCGAGCTGGACGGCGACGTAAACG
GCCACAAGTTCAGCGTGTCCGGCGAGGGCGAGGGCGATGCCACCTACGGCAAGCTGACCCTGAAGTTCATCTGCA
CCACCGGCAAGCTGCCCGTGCCCTGGCCCACCCTCGTGACCACCCTGACCTACGGCGTGCAGTGCTTCAGCCGCTA
CCCCGACCACATGAAGCAGCACGACTTCTTCAAGTCCGCCATGCCCGAAGGCTACGTCCAGGAGCGCACCATCTTC
TTCAAGGACGACGGCAACTACAAGACCCGCGCCGAGGTGAAGTTCGAGGGCGACACCCTGGTGAACCGCATCGA
GCTGAAGGGCATCGACTTCAAGGAGGACGGCAACATCCTGGGGCACAAGCTGGAGTACAACTACAACAGCCACA
ACGTCTATATCATGGCCGACAAGCAGAAGAACGGCATCAAGGTGAACTTCAAGATCCGCCACAACATCGAGGACG
GCAGCGTGCAGCTCGCCGACCACTACCAGCAGAACACCCCCATCGGCGACGGCCCCGTGCTGCTGCCCGACAACC
ACTACCTGAGCACCCAGTCCGCCCTGAGCAAAGACCCCAACGAGAAGCGCGATCACATGGTCCTGCTGGAGTTCG
TGACCGCCGCCGGGATCACTCTCGGCATGGACGAGCTGTACAAGGCCAAGCCTTTGTCTCAAGAAGAATCCACCC
TCATTGAAAGAGCAACGGCTACAATCAACAGCATCCCCATCTCTGAAGACTACAGCGTCGCCAGCGCAGCTCTCTC
TAGCGACGGCCGCATCTTCACTGGTGTCAATGTATATCATTTTACTGGGGGACCTTGTGCAGAACTCGTGGTGCTG
GGCACTGCTGCTGCTGCGGCAGCTGGCAACCTGACTTGTATCGTCGCGATCGGAAATGAGAACAGGGGCATCTTG
AGCCCCTGCGGACGGTGCCGACAGGTGCTTCTCGATCTGCATCCTGGGATCAAAGCCATAGTGAAGGACAGTGAT
GGACAGCCGACGGCAGTTGGGATTCGTGAATTGCTGCCCTCTGGTTATGTGTGGGAGGGCTAAGAATTCATGATC
GCTGATGGTGCATAAAATAGTGTACCACGTTGTTTATTTTACTCTTTATATAAGGATAACATTCTTTGTTCTTCATCG
ATAATGTGGTATAATACTGACTGCCGGTTTTATTGGTAATCATTTCGGCTCAGTTGCTTTTAGTACATCAATTTTAAT
TATTATCTGTTTTATTTCGTTTTTATAGTGTTTTAGTTTCTAGCACTTATCAAATCACGGGGTTCGGTCGCATTACACT
GCTATGTGCTTTGAAATCGGTGTTTCGTTAGGCCTAATAATTACTAGTTACTATTATATGCAACGATTAACAGATAA
TTATATCGGCAGTGGTTTGTTAAGCTTCTTGGCACGCTTTATTGCTCTGCGCCGAGCTTCCCATTCTTGATCGTGTGC
TGCAGTCTCCTTTAACTGTGCGTTCAGTCGTTCCATATATGCCTTCTTCTCTGTTGATATATCTACTAGTGTAGGTAC
CTCATCTTGATATCGCTCATCCTCAACTTCTTCATCTGAGTCTGCCTCAAACACCACATGCTTCTGTTCAGTTTGTTTG
ATTGTTTCCACACCACGTAGTTTAGCAACACCATGCCTATTTAATCGGAATTTCTTCCTTGTTGATGCTAGGTTGGCT
TTCAGCTTTACATCTTCGTTATCCTCTGTTGCCTTTTGTTCTATGTAATGTTTCAGGTCGTCATCCACTCTCGAGGCAT
CAGTTCCAACACGTGTGAGTATATTATCCGTGTCAGCGTCAGATTCATCGGACGATTCATCCGAATCCACGACAGT
ACTACTGACATCAGACTCACTTGCGATGTCCACCTTTGTTTCATTAGACAGCTGTTTCTTGGCTTTTATCTTTTCCTTT
AGCTTCGAGAGCTTCGAAGGCTTTTCGCTAGTATTGTCATTTACAGTTATTTGGGGTGCTG

Seq 8: Figure 7 A- TF acquisition (1250 bp band)

ATCGCTTGGACCTATGTTATGGTTTCTGAACGTCGTTCTGCTGCTAGCAGTGTAGTTTGTCGTCTATGTGCGAAGAC
TACTAAATGCTCTCGCAGTCGTAACCTGCAACAGGCGCATATAATGCACCTCATTCGTATGCACAAGAACCCTTGG
AAGCAGTATTTGGATGAGGAGGGACTCACTAAAATGTGTAGATCCGCTCTGGATGCTTTGTATCAGCAGCGTGGT
TTTACTGCTACAAGTGAATGGAAGTTGAGGCCGGGTCTCACTCCACCTGAGCCTGGGTTCGATTGTTCTTCAATGG
TTGATGGTGATAGTGTTTTGGATTAGATGATCGCTGATGGTGCATAAAATAGTGTACCACGTTGTTTATTTTACTCT
TTATATAAGGATAACATTCTTTGTTCTTCATCGATAATGTGGTATAATACTGACTGCCGGTTTTATTGGTAATCATTT
CGGCTCAGTTGCTTTTAGTACATCAATTTTAATTATTATCTGTTTTATTTCGTTTTTATAGTGTTTTAGTTTCTAGCACT
TATCAAATCACGGGGTTCGGTCGCATTACACTGCTATGTGCTTTGAAATCGGTGTTTCGTTAGGCCTAATAATTACT
AGTTACTATTATATGCAACGATTAACAGATAATTATATCGGCAGTGGTTTGTTAAGCTTCTTGGCACGCTTTATTGC
TCTGCGCCGAGCTTCCCATTCTTGATCGTGTGCTGCAGTCTCCTTTAACTGTGCGTTCAGTCGTTCCATATATGCCTT
CTTCTCTGTTGATATATCTACTAGTGTAGGTACCTCATCTTGATATCGCTCATCCTCAACTTCTTCATCTGAGTCTGCC
TCAAACACCACATGCTTCTGTTCAGTTTGTTTGATTGTTTCCACACCACGTAGTTTAGCAACACCATGCCTATTTAAT
CGGAATTTCTTCCTTGTTGATGCTAGGTTGGCTTTCAGCTTTACATCTTCGTTATCCTCTGTTGCCTTTTGTTCTATGT
AATGTTTCAGGTCGTCATCCACTGCATCAGTTCCAACACGTGTGAGTATATTATCCGTGTCAGCGTCAGATTCATCG
GACGATTCATCCGAATCCACGACAGTACTACTGACATCAGACTCACTTGCGATGTCCACCTTTGTTTCATTAGACAG
CTGTTTCTTGGCTTTTATCTTTTCCTTTAGCTTCGAGAGCTTCGAAGGCTTTTCGCTAGTATTGTCATTTACAGTTATT
TGGGGTGCTG

Seq 9: Figure 7 A-WT acquisition (1250 bp band)

ATCGCTTGGACCTATGTTATGGTTTCTGAACGTCGTTCTGCTGCTAGCAGTGTAGTTTGTCGTCTATGTGCGAAGAC
TACTAAATGCTCTCGCAGTCGTAACCTGCAACAGGCGCATATAATGCACCTCATTCGTATGCACAAGAACCCTTGG
AAGCAGTATTTGGATGAGGAGGGACTCACTAAAATGTGTAGATCCGCTCTGGATGCTTTGTATCAGCAGCGTGGT
TTTACTGCTACAAGTGAATGGAAGTTGAGGCCGGGTCTCACTCCACCTGAGCCTGGGTTCGATTGTTCTTCAATGG
TTGATGGTGATAGTGTTTTGGATTAGATGATCGCTGATGGTGCATAAAATAGTGTACCACGTTGTTTATTTTACTCT
TTATATAAGGATAACATTCTTTGTTCTTCATCGATAATGTGGTATAATACTGACTGCCGGTTTTATTGGTAATCATTT
CGGCTCAGTTGCTTTTAGTACATCAATTTTAATTATTATCTGTTTTATTTCGTTTTTATAGTGTTTTAGTTTCTAGCACT
TATCAAATCACGGGGTTCGGTCGCATTACACTGCTATGTGCTTTGAAATCGGTGTTTCGTTAGGCCTAATAATTACT
AGTTACTATTATATGCAACGATTAACAGATAATTATATCGGCAGTGGTTTGTTAAGCTTCTTGGCACGCTTTATTGC
TCTGCGCCGAGCTTCCCATTCTTGATCGTGTGCTGCAGTCTCCTTTAACTGTGCGTTCAGTCGTTCCATATATGCCTT
CTTCTCTGTTGATATATCTACTAGTGTAGGTACCTCATCTTGATATCGCTCATCCTCAACTTCTTCATCTGAGTCTGCC
TCAAACACCACATGCTTCTGTTCAGTTTGTTTGATTGTTTCCACACCACGTAGTTTAGCAACACCATGCCTATTTAAT
CGGAATTTCTTCCTTGTTGATGCTAGGTTGGCTTTCAGCTTTACATCTTCGTTATCCTCTGTTGCCTTTTGTTCTATGT
AATGTTTCAGGTCGTCATCCACTGCATCAGTTCCAACACGTGTGAGTATATTATCCGTGTCAGCGTCAGATTCATCG
GACGATTCATCCGAATCCACGACAGTACTACTGACATCAGACTCACTTGCGATGTCCACCTTTGTTTCATTAGACAG
CTGTTTCTTGGCTTTTATCTTTTCCTTTAGCTTCGAGAGCTTCGAAGGCTTTTCGCTAGTATTGTCATTTACAGTTATT
TGGGGTGCTG

Seq 10: Figure 7 A- TF transmission (3656 bp band)

ATCGCTTGGACCTATGTTATGGTTTCTGAACGTCGTTCTGCTGCTAGCAGTGTAGTTTGTCGTCTATGTGCGAAGAC
TACTAAATGCTCTCGCAGTCGTAACCTGCAACAGGCGCATATAATGCACCTCATTCGTATGCACAAGAACCCTTGG
AAGCAGTATTTGGATGAGGAGGGACTCACTAAAATGTGTAGATCCGCTCTGGATGCTTTGTATCAGCAGCGTGGT
TTTACTGCTACAAGTGAATGGAAGTTGAGGCCGGGTCTCACTCCACCTGAGCCTGGGTTCGATTGTTCTTCAATGG
TTGATGGTGATAGTGTTTTGGATGCTAGCATGTGCCAAGATCCTCAACGCAAAGGCAACGTGATACTCTCTCAGGC
TTACGGGTGCCAAGATCCTCAACGCAAAGGCAACGTGATACTCTCTCAGGCTTACTAGCCGCGGTCTAGAGGATCC

TAATGGCACAAACTCAACAAATGATGTATCGTCATCTGATCCATCGGTTTTCAATATTGTATTGGATGCAATATCTG
AATGCATATGATGCGACAGTTTCCATCATCGGGTGCCGAATCGTAACTCTCATAACACCATTTTAAGTTATGTAAAC
TAGTATCTATGTTTATGGTTTACGTAAAATGAATGTTTGAGTCTACAGGAAGGAACACGTGTGTAGAAGTATTTAT
TTCGTGAATGTACGTCATATATTTTTATGTATACGTGAACCTCACTAAAGGAAATTTTTGATGTTTTACGTATGCATT
TATTAAACAACACTCTGTTAAATTATTCCATACGCTAAGATATGATTCCTTTCTCGCAAATCTCCCCGGGCTGCTGGA
AAAAAATCAATTGATATATAAATAAACTTATATAAACCACGTAATAAATGAGATAAATAAATATATGTCATGTATAA
ATTTGGGGTATAATTTTAATTGTAGAAGGTGGAGATAGTACGGTAAAAACTGAGATGATATATAATATATGATGA
ATATATAAACTTGACAGGTCAAGATTACTTGATCAGATTTAATTAGTTGTGATAAAGCGGAAATTCCCATATATGTC
ATCCCGTTAAAGGTATTAATAGCATTTTAAGAATATATTTCACAAATATGATATACCATACATCTGATAGAATTATG
CATCGATATTCGTCGTTATAGGCTTAGTTGCGCACAACGAAAAGGTATGTGTATATACTTAATTATATTTCAATCAA
ACGATGGGTGACAAATGTGTGGCGTGGAGCAAAGCAAAACAGCACCACAACCTTTACAAAGGGATCTTCATTAGT
CCTTTGCAGTGTCTTTATAACTTAATAAAGTAATTCCACGCAAGAATAGTGATTATATCGCCAACAACGCACTTTTG
TGATGTAAGTCAGGCCCCTGTCATTTTGCTTTGTGGGTGGTGTTATTTCATGAAGCAGGCGACTACTTCTTGTCGAG
CTGCTGTGTGTATATTCTACTTTTGAGGCTATTATATACACTGATGTGTTTAGACATTTGTGTTATTTAATGCTTGAT
TTAACGTTTTTTCAATTGTGTTTGCCGATACTAATGTTGTCTCAGATATAATTTATTGCAACTTTACGAAAATAAGCT
TATGGTGAGCAAGGGCGAGGAGCTGTTCACCGGGGTGGTGCCCATCCTGGTCGAGCTGGACGGCGACGTAAACG
GCCACAAGTTCAGCGTGTCCGGCGAGGGCGAGGGCGATGCCACCTACGGCAAGCTGACCCTGAAGTTCATCTGCA
CCACCGGCAAGCTGCCCGTGCCCTGGCCCACCCTCGTGACCACCCTGACCTACGGCGTGCAGTGCTTCAGCCGCTA
CCCCGACCACATGAAGCAGCACGACTTCTTCAAGTCCGCCATGCCCGAAGGCTACGTCCAGGAGCGCACCATCTTC
TTCAAGGACGACGGCAACTACAAGACCCGCGCCGAGGTGAAGTTCGAGGGCGACACCCTGGTGAACCGCATCGA
GCTGAAGGGCATCGACTTCAAGGAGGACGGCAACATCCTGGGGCACAAGCTGGAGTACAACTACAACAGCCACA
ACGTCTATATCATGGCCGACAAGCAGAAGAACGGCATCAAGGTGAACTTCAAGATCCGCCACAACATCGAGGACG
GCAGCGTGCAGCTCGCCGACCACTACCAGCAGAACACCCCCATCGGCGACGGCCCCGTGCTGCTGCCCGACAACC
ACTACCTGAGCACCCAGTCCGCCCTGAGCAAAGACCCCAACGAGAAGCGCGATCACATGGTCCTGCTGGAGTTCG
TGACCGCCGCCGGGATCACTCTCGGCATGGACGAGCTGTACAAGGCCAAGCCTTTGTCTCAAGAAGAATCCACCC
TCATTGAAAGAGCAACGGCTACAATCAACAGCATCCCCATCTCTGAAGACTACAGCGTCGCCAGCGCAGCTCTCTC
TAGCGACGGCCGCATCTTCACTGGTGTCAATGTATATCATTTTACTGGGGGACCTTGTGCAGAACTCGTGGTGCTG
GGCACTGCTGCTGCTGCGGCAGCTGGCAACCTGACTTGTATCGTCGCGATCGGAAATGAGAACAGGGGCATCTTG
AGCCCCTGCGGACGGTGCCGACAGGTGCTTCTCGATCTGCATCCTGGGATCAAAGCCATAGTGAAGGACAGTGAT
GGACAGCCGACGGCAGTTGGGATTCGTGAATTGCTGCCCTCTGGTTATGTGTGGGAGGGCTAAGAATTCATGATC
GCTGATGGTGCATAAAATAGTGTACCACGTTGTTTATTTTACTCTTTATATAAGGATAACATTCTTTGTTCTTCATCG
ATAATGTGGTATAATACTGACTGCCGGTTTTATTGGTAATCATTTCGGCTCAGTTGCTTTTAGTACATCAATTTTAAT
TATTATCTGTTTTATTTCGTTTTTATAGTGTTTTAGTTTCTAGCACTTATCAAATCACGGGGTTCGGTCGCATTACACT
GCTATGTGCTTTGAAATCGGTGTTTCGTTAGGCCTAATAATTACTAGTTACTATTATATGCAACGATTAACAGATAA
TTATATCGGCAGTGGTTTGTTAAGCTTCTTGGCACGCTTTATTGCTCTGCGCCGAGCTTCCCATTCTTGATCGTGTGC
TGCAGTCTCCTTTAACTGTGCGTTCAGTCGTTCCATATATGCCTTCTTCTCTGTTGATATATCTACTAGTGTAGGTAC
CTCATCTTGATATCGCTCATCCTCAACTTCTTCATCTGAGTCTGCCTCAAACACCACATGCTTCTGTTCAGTTTGTTTG
ATTGTTTCCACACCACGTAGTTTAGCAACACCATGCCTATTTAATCGGAATTTCTTCCTTGTTGATGCTAGGTTGGCT
TTCAGCTTTACATCTTCGTTATCCTCTGTTGCCTTTTGTTCTATGTAATGTTTCAGGTCGTCATCCACTCTCGAGGCAT
CAGTTCCAACACGTGTGAGTATATTATCCGTGTCAGCGTCAGATTCATCGGACGATTCATCCGAATCCACGACAGT
ACTACTGACATCAGACTCACTTGCGATGTCCACCTTTGTTTCATTAGACAGCTGTTTCTTGGCTTTTATCTTTTCCTTT
AGCTTCGAGAGCTTCGAAGGCTTTTCGCTAGTATTGTCATTTACAGTTATTTGGGGTGCTG

Seq 11: Figure 7 A- TF transmission (1250 bp band)

ATCGCTTGGACCTATGTTATGGTTTCTGAACGTCGTTCTGCTGCTAGCAGTGTAGTTTGTCGTCTATGTGCGAAGAC
TACTAAATGCTCTCGCAGTCGTAACCTGCAACAGGCGCATATAATGCACCTCATTCGTATGCACAAGAACCCTTGG
AAGCAGTATTTGGATGAGGAGGGACTCACTAAAATGTGTAGATCCGCTCTGGATGCTTTGTATCAGCAGCGTGGT
TTTACTGCTACAAGTGAATGGAAGTTGAGGCCGGGTCTCACTCCACCTGAGCCTGGGTTCGATTGTTCTTCAATGG
TTGATGGTGATAGTGTTTTGGATTAGATGATCGCTGATGGTGCATAAAATAGTGTACCACGTTGTTTATTTTACTCT
TTATATAAGGATAACATTCTTTGTTCTTCATCGATAATGTGGTATAATACTGACTGCCGGTTTTATTGGTAATCATTT
CGGCTCAGTTGCTTTTAGTACATCAATTTTAATTATTATCTGTTTTATTTCGTTTTTATAGTGTTTTAGTTTCTAGCACT
TATCAAATCACGGGGTTCGGTCGCATTACACTGCTATGTGCTTTGAAATCGGTGTTTCGTTAGGCCTAATAATTACT
AGTTACTATTATATGCAACGATTAACAGATAATTATATCGGCAGTGGTTTGTTAAGCTTCTTGGCACGCTTTATTGC
TCTGCGCCGAGCTTCCCATTCTTGATCGTGTGCTGCAGTCTCCTTTAACTGTGCGTTCAGTCGTTCCATATATGCCTT
CTTCTCTGTTGATATATCTACTAGTGTAGGTACCTCATCTTGATATCGCTCATCCTCAACTTCTTCATCTGAGTCTGCC
TCAAACACCACATGCTTCTGTTCAGTTTGTTTGATTGTTTCCACACCACGTAGTTTAGCAACACCATGCCTATTTAAT
CGGAATTTCTTCCTTGTTGATGCTAGGTTGGCTTTCAGCTTTACATCTTCGTTATCCTCTGTTGCCTTTTGTTCTATGT
AATGTTTCAGGTCGTCATCCACTGCATCAGTTCCAACACGTGTGAGTATATTATCCGTGTCAGCGTCAGATTCATCG
GACGATTCATCCGAATCCACGACAGTACTACTGACATCAGACTCACTTGCGATGTCCACCTTTGTTTCATTAGACAG
CTGTTTCTTGGCTTTTATCTTTTCCTTTAGCTTCGAGAGCTTCGAAGGCTTTTCGCTAGTATTGTCATTTACAGTTATT
TGGGGTGCTG

Seq 12: Figure 7 A-WT transmission (d -1250 bp)

ATCGCTTGGACCTATGTTATGGTTTCTGAACGTCGTTCTGCTGCTAGCAGTGTAGTTTGTCGTCTATGTGCGAAGAC
TACTAAATGCTCTCGCAGTCGTAACCTGCAACAGGCGCATATAATGCACCTCATTCGTATGCACAAGAACCCTTGG
AAGCAGTATTTGGATGAGGAGGGACTCACTAAAATGTGTAGATCCGCTCTGGATGCTTTGTATCAGCAGCGTGGT
TTTACTGCTACAAGTGAATGGAAGTTGAGGCCGGGTCTCACTCCACCTGAGCCTGGGTTCGATTGTTCTTCAATGG
TTGATGGTGATAGTGTTTTGGATTAGATGATCGCTGATGGTGCATAAAATAGTGTACCACGTTGTTTATTTTACTCT
TTATATAAGGATAACATTCTTTGTTCTTCATCGATAATGTGGTATAATACTGACTGCCGGTTTTATTGGTAATCATTT
CGGCTCAGTTGCTTTTAGTACATCAATTTTAATTATTATCTGTTTTATTTCGTTTTTATAGTGTTTTAGTTTCTAGCACT
TATCAAATCACGGGGTTCGGTCGCATTACACTGCTATGTGCTTTGAAATCGGTGTTTCGTTAGGCCTAATAATTACT
AGTTACTATTATATGCAACGATTAACAGATAATTATATCGGCAGTGGTTTGTTAAGCTTCTTGGCACGCTTTATTGC
TCTGCGCCGAGCTTCCCATTCTTGATCGTGTGCTGCAGTCTCCTTTAACTGTGCGTTCAGTCGTTCCATATATGCCTT
CTTCTCTGTTGATATATCTACTAGTGTAGGTACCTCATCTTGATATCGCTCATCCTCAACTTCTTCATCTGAGTCTGCC
TCAAACACCACATGCTTCTGTTCAGTTTGTTTGATTGTTTCCACACCACGTAGTTTAGCAACACCATGCCTATTTAAT
CGGAATTTCTTCCTTGTTGATGCTAGGTTGGCTTTCAGCTTTACATCTTCGTTATCCTCTGTTGCCTTTTGTTCTATGT
AATGTTTCAGGTCGTCATCCACTGCATCAGTTCCAACACGTGTGAGTATATTATCCGTGTCAGCGTCAGATTCATCG
GACGATTCATCCGAATCCACGACAGTACTACTGACATCAGACTCACTTGCGATGTCCACCTTTGTTTCATTAGACAG
CTGTTTCTTGGCTTTTATCTTTTCCTTTAGCTTCGAGAGCTTCGAAGGCTTTTCGCTAGTATTGTCATTTACAGTTATT
TGGGGTGCTG

Seq 13: Figure 7 B1- TF acquisition (1551 bp band)

ATGTCACTGACTGCTCAGCCCGAACACGTTGCACAGGAAGTGCCTACTCACGTTATTCCCTATTCTGAGAACTACAT
GGGATATTACCCTGGTGATATGAGTGCAGGTCTACAATGTCACGGTTACCTGCCTTCTCCTCAATACACTCAATATT
ATGATGACCCTTACATGGGTACAGCGGTTGACTACATGAACGTCACCTGTTACCAGATGGTCCCAGATGACCATCA
TGGTTACGTCGACTCTCATGTTGGACAAATGCCCGACTACTCCGGTGGCATGATGTACCATCCTATGGAGATGACA

TACTGCCCTCCTACTGGGCAGATGTACTATCAAACACCTGGATCGAGTACCGTACCGGCATTAAGCAGCGTCAAGG
GGGATCCCGATGACGATGCTCGTACTTTTACTGAATGCAGTACTGCGGCAACTACTCCTAACTTGGAACACTGCCC
CAACCCCGAAGTCGTTGAAGATGGATTTGATCTTGGTTATGGATACGACTACGACCGATACGCAGTATCACCTTCT
GACTGCTACCCTCGCCAAAGTGCTGGGTTTAGCGGTTACCCTGGTTACTGTGTGTCCAACTTTGACGATGTATTTTC
CGCTTGTGGCGACCAGGACCCTTTAGTGAACGATGCTATGGTTGTGGAGATTGCTGACGAGATAGCGGCCAACGT
CCAGTATTTACCAGACAAGGAATCCAGTGGTAAATACTCACTTGACAAAAACCACCCGATTCATTGCGTCTGGCGT
GATCTCAACAGAGGTCACTGCAGTTGGCGTTGCCGCTGGTGGGAGAACGGCAAGCGTTTGAGTAAGAACTTCAAC
GTGAAACGCTTCGGTGAGCATGAGGCCATGTGGATGGCCATTGCTATGAAGATTCGTAACTCTACGCCTGTTGAG
CGTTTTCAGTTACTCAAGGAGCAGCGTGAAGCTGTTCGCAACTACTGTGAGCTTGTGCGTCAAAACGTTGATGGGT
CGGTTGTTGACTATCGTGTTGATCCAAAAAGTTCTATTGCTATAGCTCAGATTGGCACTGCTACGCGCAAGGCCCG
ATCACCTCTGAACAGCACTAAGAGTGCTCAGAAGGCCAACTGGGCGCGCATCGCTTGGACCTATGTTATGGTTTCT
GAACGTCGTTCTGCTGCTAGCAGTGTAGTTTGTCGTCTATGTGCGAAGACTACTAAATGCTCTCGCAGTCGTAACCT
GCAACAGGCGCATATAATGCACCTCATTCGTATGCACAAGAACCCTTGGAAGCAGTATTTGGATGAGGAGGGACT
CACTAAAATGTGTAGATCCGCTCTGGATGCTTTGTATCAGCAGCGTGGTTTTACTGCTACAAGTGAATGGAAGTTG
AGGCCGGGTCTCACTCCACCTGAGCCTGGGTTCGATTGTTCTTCAATGGTTGATGGTGATAGTGTTTTGGATGCTA
GCATGTGCCAAGATCCTCAACGCAAAGGCAACGTGATACTCTCTCAGGCTTACGGGTGCCAAGATCCTCAACGCAA
AGGCAACGTGATACTCTCTCAGGCTTACTAG

Seq 14: Figure 7 B1 - TF transmission (1551 bp band)

ATGTCACTGACTGCTCAGCCCGAACACGTTGCACAGGAAGTGCCTACTCACGTTATTCCCTATTCTGAGAACTACAT
GGGATATTACCCTGGTGATATGAGTGCAGGTCTACAATGTCACGGTTACCTGCCTTCTCCTCAATACACTCAATATT
ATGATGACCCTTACATGGGTACAGCGGTTGACTACATGAACGTCACCTGTTACCAGATGGTCCCAGATGACCATCA
TGGTTACGTCGACTCTCATGTTGGACAAATGCCCGACTACTCCGGTGGCATGATGTACCATCCTATGGAGATGACA
TACTGCCCTCCTACTGGGCAGATGTACTATCAAACACCTGGATCGAGTACCGTACCGGCATTAAGCAGCGTCAAGG
GGGATCCCGATGACGATGCTCGTACTTTTACTGAATGCAGTACTGCGGCAACTACTCCTAACTTGGAACACTGCCC
CAACCCCGAAGTCGTTGAAGATGGATTTGATCTTGGTTATGGATACGACTACGACCGATACGCAGTATCACCTTCT
GACTGCTACCCTCGCCAAAGTGCTGGGTTTAGCGGTTACCCTGGTTACTGTGTGTCCAACTTTGACGATGTATTTTC
CGCTTGTGGCGACCAGGACCCTTTAGTGAACGATGCTATGGTTGTGGAGATTGCTGACGAGATAGCGGCCAACGT
CCAGTATTTACCAGACAAGGAATCCAGTGGTAAATACTCACTTGACAAAAACCACCCGATTCATTGCGTCTGGCGT
GATCTCAACAGAGGTCACTGCAGTTGGCGTTGCCGCTGGTGGGAGAACGGCAAGCGTTTGAGTAAGAACTTCAAC
GTGAAACGCTTCGGTGAGCATGAGGCCATGTGGATGGCCATTGCTATGAAGATTCGTAACTCTACGCCTGTTGAG
CGTTTTCAGTTACTCAAGGAGCAGCGTGAAGCTGTTCGCAACTACTGTGAGCTTGTGCGTCAAAACGTTGATGGGT
CGGTTGTTGACTATCGTGTTGATCCAAAAAGTTCTATTGCTATAGCTCAGATTGGCACTGCTACGCGCAAGGCCCG
ATCACCTCTGAACAGCACTAAGAGTGCTCAGAAGGCCAACTGGGCGCGCATCGCTTGGACCTATGTTATGGTTTCT
GAACGTCGTTCTGCTGCTAGCAGTGTAGTTTGTCGTCTATGTGCGAAGACTACTAAATGCTCTCGCAGTCGTAACCT
GCAACAGGCGCATATAATGCACCTCATTCGTATGCACAAGAACCCTTGGAAGCAGTATTTGGATGAGGAGGGACT
CACTAAAATGTGTAGATCCGCTCTGGATGCTTTGTATCAGCAGCGTGGTTTTACTGCTACAAGTGAATGGAAGTTG
AGGCCGGGTCTCACTCCACCTGAGCCTGGGTTCGATTGTTCTTCAATGGTTGATGGTGATAGTGTTTTGGATGCTA
GCATGTGCCAAGATCCTCAACGCAAAGGCAACGTGATACTCTCTCAGGCTTACGGGTGCCAAGATCCTCAACGCAA
AGGCAACGTGATACTCTCTCAGGCTTACTAG

Seq 15: Figure 7 B2- TF acquisition (1109 bp band)

ATGGTGAGCAAGGGCGAGGAGCTGTTCACCGGGGTGGTGCCCATCCTGGTCGAGCTGGACGGCGACGTAAACG
GCCACAAGTTCAGCGTGTCCGGCGAGGGCGAGGGCGATGCCACCTACGGCAAGCTGACCCTGAAGTTCATCTGCA
CCACCGGCAAGCTGCCCGTGCCCTGGCCCACCCTCGTGACCACCCTGACCTACGGCGTGCAGTGCTTCAGCCGCTA
CCCCGACCACATGAAGCAGCACGACTTCTTCAAGTCCGCCATGCCCGAAGGCTACGTCCAGGAGCGCACCATCTTC
TTCAAGGACGACGGCAACTACAAGACCCGCGCCGAGGTGAAGTTCGAGGGCGACACCCTGGTGAACCGCATCGA
GCTGAAGGGCATCGACTTCAAGGAGGACGGCAACATCCTGGGGCACAAGCTGGAGTACAACTACAACAGCCACA
ACGTCTATATCATGGCCGACAAGCAGAAGAACGGCATCAAGGTGAACTTCAAGATCCGCCACAACATCGAGGACG
GCAGCGTGCAGCTCGCCGACCACTACCAGCAGAACACCCCCATCGGCGACGGCCCCGTGCTGCTGCCCGACAACC
ACTACCTGAGCACCCAGTCCGCCCTGAGCAAAGACCCCAACGAGAAGCGCGATCACATGGTCCTGCTGGAGTTCG
TGACCGCCGCCGGGATCACTCTCGGCATGGACGAGCTGTACAAGGCCAAGCCTTTGTCTCAAGAAGAATCCACCC
TCATTGAAAGAGCAACGGCTACAATCAACAGCATCCCCATCTCTGAAGACTACAGCGTCGCCAGCGCAGCTCTCTC
TAGCGACGGCCGCATCTTCACTGGTGTCAATGTATATCATTTTACTGGGGGACCTTGTGCAGAACTCGTGGTGCTG
GGCACTGCTGCTGCTGCGGCAGCTGGCAACCTGACTTGTATCGTCGCGATCGGAAATGAGAACAGGGGCATCTTG
AGCCCCTGCGGACGGTGCCGACAGGTGCTTCTCGATCTGCATCCTGGGATCAAAGCCATAGTGAAGGACAGTGAT
GGACAGCCGACGGCAGTTGGGATTCGTGAATTGCTGCCCTCTGGTTATGTGTGGGAGGG

Seq 16: Figure 7 B2- TF transmission (1109 bp band)

ATGGTGAGCAAGGGCGAGGAGCTGTTCACCGGGGTGGTGCCCATCCTGGTCGAGCTGGACGGCGACGTAAACG
GCCACAAGTTCAGCGTGTCCGGCGAGGGCGAGGGCGATGCCACCTACGGCAAGCTGACCCTGAAGTTCATCTGCA
CCACCGGCAAGCTGCCCGTGCCCTGGCCCACCCTCGTGACCACCCTGACCTACGGCGTGCAGTGCTTCAGCCGCTA
CCCCGACCACATGAAGCAGCACGACTTCTTCAAGTCCGCCATGCCCGAAGGCTACGTCCAGGAGCGCACCATCTTC
TTCAAGGACGACGGCAACTACAAGACCCGCGCCGAGGTGAAGTTCGAGGGCGACACCCTGGTGAACCGCATCGA
GCTGAAGGGCATCGACTTCAAGGAGGACGGCAACATCCTGGGGCACAAGCTGGAGTACAACTACAACAGCCACA
ACGTCTATATCATGGCCGACAAGCAGAAGAACGGCATCAAGGTGAACTTCAAGATCCGCCACAACATCGAGGACG
GCAGCGTGCAGCTCGCCGACCACTACCAGCAGAACACCCCCATCGGCGACGGCCCCGTGCTGCTGCCCGACAACC
ACTACCTGAGCACCCAGTCCGCCCTGAGCAAAGACCCCAACGAGAAGCGCGATCACATGGTCCTGCTGGAGTTCG
TGACCGCCGCCGGGATCACTCTCGGCATGGACGAGCTGTACAAGGCCAAGCCTTTGTCTCAAGAAGAATCCACCC
TCATTGAAAGAGCAACGGCTACAATCAACAGCATCCCCATCTCTGAAGACTACAGCGTCGCCAGCGCAGCTCTCTC
TAGCGACGGCCGCATCTTCACTGGTGTCAATGTATATCATTTTACTGGGGGACCTTGTGCAGAACTCGTGGTGCTG
GGCACTGCTGCTGCTGCGGCAGCTGGCAACCTGACTTGTATCGTCGCGATCGGAAATGAGAACAGGGGCATCTTG
AGCCCCTGCGGACGGTGCCGACAGGTGCTTCTCGATCTGCATCCTGGGATCAAAGCCATAGTGAAGGACAGTGAT
GGACAGCCGACGGCAGTTGGGATTCGTGAATTGCTGCCCTCTGGTTATGTGTGGGAGGG

Seq 17: Figure 7 B3- TF acquisition (1440 bp band)

ATGTCACTGACTGCTCAGCCCGAACACGTTGCACAGGAAGTGCCTACTCACGTTATTCCCTATTCTGAGAACTACAT
GGGATATTACCCTGGTGATATGAGTGCAGGTCTACAATGTCACGGTTACCTGCCTTCTCCTCAATACACTCAATATT
ATGATGACCCTTACATGGGTACAGCGGTTGACTACATGAACGTCACCTGTTACCAGATGGTACCGGATGACCATCA
TGGTTACGTCGACTCTCATGTTGGACAAATGCCCGACTACTCCGGTGGCATGATGTACCATCCTATGGAGATGACA
TACTGCCCTCCTACTGGGCAGATGTACTATCAAACACCTGGATCGAGTACCGTACCGGCATTAAGCAGCGTCAAGG
GGGATCCCGATGACGATGCTCGTACTTTTACTGAATGCAGTACTGCGGCAACTACTCCTAACTTGGAACACTGCCC
CAACCCCGAAGTCGTTGAAGATGGATTTGATCTTGGTTATGGATACGACTACGACCGATACGCAGTATCACCTTCT

GACTGCTACCCTCGCCAAAGTGCTGGGTTTAGCGGTTACCCTGGTTACTGTGTGTCCAACTTTGACGATGTATTTTC
CGCTTGTGGCGACCAGGACCCTTTAGTGAACGATGCTATGGTTGTGGAGATTGCTGACGAGATAGCGGCCAACGT
CCAGTATTTACCAGACAAGGAATCCAGTGGTAAATACTCACTTGACAAAAACCACCCGATTCATTGCGTCTGGCGT
GATCTCAACAGAGGTCACTGCAGTTGGCGTTGCCGCTGGTGGGAGAACGGCAAGCGTTTGAGTAAGAACTTCAAC
GTGAAACGCTTCGGTGAGCATGAGGCCATGTGGATGGCCATTGCTATGAAGATTCGTAACTCTACGCCTGTTGAG
CGTTTTCAGTTACTCAAGGAGCAGCGTGAAGCTGTTCGCAACTACTGTGAGCTTGTGCGTCAAAACGTTGATGGGT
CGGTTGTTGACTATCGTGTTGATCCAAAGAGCTCTATTGCTATAGCTCAGATTGGCACTGCTACGCGCAAGGCCCG
ATCACCTCTGAACAGCACTAAGAGTGCTCAGAAGGCCAACTGGGCGCGCATCGCTTGGACCTATGTTATGGTTTCT
GAACGTCGTTCTGCTGCTAGCAGTGTAGTTTGTCGTCTATGTGCGAAGACTACTAAATGCTCTCGCAGTCGTAACCT
GCAACAGGCGCATATAATGCACCTCATTCGTATGCACAAGAACCCTTGGAAGCAGTATTTGGATGAGGAGGGACT
CACTAAAATGTGTAGATCCGCTCTGGATGCTTTGTATCAGCAGCGTGGTTTTACTGCTACAAGTGAATGGAAGTTG
AGGCCGGGTCTCACTCCACCTGAGCCTGGGTTCGATTGTTCTTCAATGGTTGATGGTGATAGTGTTTTGGAT

Seq 18: Figure 7 B3- WT acquisition (1440 bp band)

ATGTCACTGACTGCTCAGCCCGAACACGTTGCACAGGAAGTGCCTACTCACGTTATTCCCTATTCTGAGAACTACAT
GGGATATTACCCTGGTGATATGAGTGCAGGTCTACAATGTCACGGTTACCTGCCTTCTCCTCAATACACTCAATATT
ATGATGACCCTTACATGGGTACAGCGGTTGACTACATGAACGTCACCTGTTACCAGATGGTACCGGATGACCATCA
TGGTTACGTCGACTCTCATGTTGGACAAATGCCCGACTACTCCGGTGGCATGATGTACCATCCTATGGAGATGACA
TACTGCCCTCCTACTGGGCAGATGTACTATCAAACACCTGGATCGAGTACCGTACCGGCATTAAGCAGCGTCAAGG
GGGATCCCGATGACGATGCTCGTACTTTTACTGAATGCAGTACTGCGGCAACTACTCCTAACTTGGAACACTGCCC
CAACCCCGAAGTCGTTGAAGATGGATTTGATCTTGGTTATGGATACGACTACGACCGATACGCAGTATCACCTTCT
GACTGCTACCCTCGCCAAAGTGCTGGGTTTAGCGGTTACCCTGGTTACTGTGTGTCCAACTTTGACGATGTATTTTC
CGCTTGTGGCGACCAGGACCCTTTAGTGAACGATGCTATGGTTGTGGAGATTGCTGACGAGATAGCGGCCAACGT
CCAGTATTTACCAGACAAGGAATCCAGTGGTAAATACTCACTTGACAAAAACCACCCGATTCATTGCGTCTGGCGT
GATCTCAACAGAGGTCACTGCAGTTGGCGTTGCCGCTGGTGGGAGAACGGCAAGCGTTTGAGTAAGAACTTCAAC
GTGAAACGCTTCGGTGAGCATGAGGCCATGTGGATGGCCATTGCTATGAAGATTCGTAACTCTACGCCTGTTGAG
CGTTTTCAGTTACTCAAGGAGCAGCGTGAAGCTGTTCGCAACTACTGTGAGCTTGTGCGTCAAAACGTTGATGGGT
CGGTTGTTGACTATCGTGTTGATCCAAAGAGCTCTATTGCTATAGCTCAGATTGGCACTGCTACGCGCAAGGCCCG
ATCACCTCTGAACAGCACTAAGAGTGCTCAGAAGGCCAACTGGGCGCGCATCGCTTGGACCTATGTTATGGTTTCT
GAACGTCGTTCTGCTGCTAGCAGTGTAGTTTGTCGTCTATGTGCGAAGACTACTAAATGCTCTCGCAGTCGTAACCT
GCAACAGGCGCATATAATGCACCTCATTCGTATGCACAAGAACCCTTGGAAGCAGTATTTGGATGAGGAGGGACT
CACTAAAATGTGTAGATCCGCTCTGGATGCTTTGTATCAGCAGCGTGGTTTTACTGCTACAAGTGAATGGAAGTTG
AGGCCGGGTCTCACTCCACCTGAGCCTGGGTTCGATTGTTCTTCAATGGTTGATGGTGATAGTGTTTTGGAT

Seq 19: Figure 7 B3- TF transmission (1440 bp band)

ATGTCACTGACTGCTCAGCCCGAACACGTTGCACAGGAAGTGCCTACTCACGTTATTCCCTATTCTGAGAACTACAT
GGGATATTACCCTGGTGATATGAGTGCAGGTCTACAATGTCACGGTTACCTGCCTTCTCCTCAATACACTCAATATT
ATGATGACCCTTACATGGGTACAGCGGTTGACTACATGAACGTCACCTGTTACCAGATGGTACCGGATGACCATCA
TGGTTACGTCGACTCTCATGTTGGACAAATGCCCGACTACTCCGGTGGCATGATGTACCATCCTATGGAGATGACA
TACTGCCCTCCTACTGGGCAGATGTACTATCAAACACCTGGATCGAGTACCGTACCGGCATTAAGCAGCGTCAAGG
GGGATCCCGATGACGATGCTCGTACTTTTACTGAATGCAGTACTGCGGCAACTACTCCTAACTTGGAACACTGCCC
CAACCCCGAAGTCGTTGAAGATGGATTTGATCTTGGTTATGGATACGACTACGACCGATACGCAGTATCACCTTCT
GACTGCTACCCTCGCCAAAGTGCTGGGTTTAGCGGTTACCCTGGTTACTGTGTGTCCAACTTTGACGATGTATTTTC
CGCTTGTGGCGACCAGGACCCTTTAGTGAACGATGCTATGGTTGTGGAGATTGCTGACGAGATAGCGGCCAACGT

CCAGTATTTACCAGACAAGGAATCCAGTGGTAAATACTCACTTGACAAAAACCACCCGATTCATTGCGTCTGGCGT
GATCTCAACAGAGGTCACTGCAGTTGGCGTTGCCGCTGGTGGGAGAACGGCAAGCGTTTGAGTAAGAACTTCAAC
GTGAAACGCTTCGGTGAGCATGAGGCCATGTGGATGGCCATTGCTATGAAGATTCGTAACTCTACGCCTGTTGAG
CGTTTTCAGTTACTCAAGGAGCAGCGTGAAGCTGTTCGCAACTACTGTGAGCTTGTGCGTCAAAACGTTGATGGGT
CGGTTGTTGACTATCGTGTTGATCCAAAGAGCTCTATTGCTATAGCTCAGATTGGCACTGCTACGCGCAAGGCCCG
ATCACCTCTGAACAGCACTAAGAGTGCTCAGAAGGCCAACTGGGCGCGCATCGCTTGGACCTATGTTATGGTTTCT
GAACGTCGTTCTGCTGCTAGCAGTGTAGTTTGTCGTCTATGTGCGAAGACTACTAAATGCTCTCGCAGTCGTAACCT
GCAACAGGCGCATATAATGCACCTCATTCGTATGCACAAGAACCCTTGGAAGCAGTATTTGGATGAGGAGGGACT
CACTAAAATGTGTAGATCCGCTCTGGATGCTTTGTATCAGCAGCGTGGTTTTACTGCTACAAGTGAATGGAAGTTG
AGGCCGGGTCTCACTCCACCTGAGCCTGGGTTCGATTGTTCTTCAATGGTTGATGGTGATAGTGTTTTGGAT

Seq 20: Figure 7 B3- WT transmission (1440 bp band)

ATGTCACTGACTGCTCAGCCCGAACACGTTGCACAGGAAGTGCCTACTCACGTTATTCCCTATTCTGAGAACTACAT
GGGATATTACCCTGGTGATATGAGTGCAGGTCTACAATGTCACGGTTACCTGCCTTCTCCTCAATACACTCAATATT
ATGATGACCCTTACATGGGTACAGCGGTTGACTACATGAACGTCACCTGTTACCAGATGGTACCGGATGACCATCA
TGGTTACGTCGACTCTCATGTTGGACAAATGCCCGACTACTCCGGTGGCATGATGTACCATCCTATGGAGATGACA
TACTGCCCTCCTACTGGGCAGATGTACTATCAAACACCTGGATCGAGTACCGTACCGGCATTAAGCAGCGTCAAGG
GGGATCCCGATGACGATGCTCGTACTTTTACTGAATGCAGTACTGCGGCAACTACTCCTAACTTGGAACACTGCCC
CAACCCCGAAGTCGTTGAAGATGGATTTGATCTTGGTTATGGATACGACTACGACCGATACGCAGTATCACCTTCT
GACTGCTACCCTCGCCAAAGTGCTGGGTTTAGCGGTTACCCTGGTTACTGTGTGTCCAACTTTGACGATGTATTTTC
CGCTTGTGGCGACCAGGACCCTTTAGTGAACGATGCTATGGTTGTGGAGATTGCTGACGAGATAGCGGCCAACGT
CCAGTATTTACCAGACAAGGAATCCAGTGGTAAATACTCACTTGACAAAAACCACCCGATTCATTGCGTCTGGCGT
GATCTCAACAGAGGTCACTGCAGTTGGCGTTGCCGCTGGTGGGAGAACGGCAAGCGTTTGAGTAAGAACTTCAAC
GTGAAACGCTTCGGTGAGCATGAGGCCATGTGGATGGCCATTGCTATGAAGATTCGTAACTCTACGCCTGTTGAG
CGTTTTCAGTTACTCAAGGAGCAGCGTGAAGCTGTTCGCAACTACTGTGAGCTTGTGCGTCAAAACGTTGATGGGT
CGGTTGTTGACTATCGTGTTGATCCAAAGAGCTCTATTGCTATAGCTCAGATTGGCACTGCTACGCGCAAGGCCCG
ATCACCTCTGAACAGCACTAAGAGTGCTCAGAAGGCCAACTGGGCGCGCATCGCTTGGACCTATGTTATGGTTTCT
GAACGTCGTTCTGCTGCTAGCAGTGTAGTTTGTCGTCTATGTGCGAAGACTACTAAATGCTCTCGCAGTCGTAACCT
GCAACAGGCGCATATAATGCACCTCATTCGTATGCACAAGAACCCTTGGAAGCAGTATTTGGATGAGGAGGGACT
CACTAAAATGTGTAGATCCGCTCTGGATGCTTTGTATCAGCAGCGTGGTTTTACTGCTACAAGTGAATGGAAGTTG
AGGCCGGGTCTCACTCCACCTGAGCCTGGGTTCGATTGTTCTTCAATGGTTGATGGTGATAGTGTTTTGGAT

Seq 21: Figure 7 B4- TF acquisition (350 bp band)

CACGAGGAAGGAACTACCGATGTTGAATATCTTGTCAACAAGGTACTCTATATGGCTACCATGAACTACAAGACTT
ATTTGACAGTAAACAGTATGAACGCCAAGTTCTTCAACAGATTCAGCTTCACTACAAAGATATTCAGTCGTCGTATT
AAGCAAACATTGAGTGATATCATCAGGTGGAATGTTCCTGAAGATTTTGAAGAAAGGAGCATCGAACGTATCACT
CACTTACTAGCAGCTACGAAGATTACATGTTGACCCAGATTCCAACTCTTTCCAAGTTTGCACGTCGTTATGCTGAC
ATGGTGAAGAAGGTTCTGCTCGGTAGCTTGACCTCGTACGTTGAAGCTCCTTGG

Seq 22: Figure 7 B4- WT acquisition (350 bp band)

CACGAGGAAGGAACTACCGATGTTGAATATCTTGTCAACAAGGTACTCTATATGGCTACCATGAACTACAAGACTT
ATTTGACAGTAAACAGTATGAACGCCAAGTTCTTCAACAGATTCAGCTTCACTACAAAGATATTCAGTCGTCGTATT
AAGCAAACATTGAGTGATATCATCAGGTGGAATGTTCCTGAAGATTTTGAAGAAAGGAGCATCGAACGTATCACT

CACTTACTAGCAGCTACGAAGATTACATGTTGACCCAGATTCCAACTCTTTCCAAGTTTGCACGTCGTTATGCTGAC
ATGGTGAAGAAGGTTCTGCTCGGTAGCTTGACCTCGTACGTTGAAGCTCCTTGG

Seq 23: Figure 7 B4- TF acquisition (350 bp band)

CACGAGGAAGGAACTACCGATGTTGAATATCTTGTCAACAAGGTACTCTATATGGCTACCATGAACTACAAGACTT
ATTTGACAGTAAACAGTATGAACGCCAAGTTCTTCAACAGATTCAGCTTCACTACAAAGATATTCAGTCGTCGTATT
AAGCAAACATTGAGTGATATCATCAGGTGGAATGTTCCTGAAGATTTTGAAGAAAGGAGCATCGAACGTATCACT
CACTTACTAGCAGCTACGAAGATTACATGTTGACCCAGATTCCAACTCTTTCCAAGTTTGCACGTCGTTATGCTGAC
ATGGTGAAGAAGGTTCTGCTCGGTAGCTTGACCTCGTACGTTGAAGCTCCTTGG

Seq 24: Figure 7 B4- WT transmission (350 bp band)

CACGAGGAAGGAACTACCGATGTTGAATATCTTGTCAACAAGGTACTCTATATGGCTACCATGAACTACAAGACTT
ATTTGACAGTAAACAGTATGAACGCCAAGTTCTTCAACAGATTCAGCTTCACTACAAAGATATTCAGTCGTCGTATT
AAGCAAACATTGAGTGATATCATCAGGTGGAATGTTCCTGAAGATTTTGAAGAAAGGAGCATCGAACGTATCACT
CACTTACTAGCAGCTACGAAGATTACATGTTGACCCAGATTCCAACTCTTTCCAAGTTTGCACGTCGTTATGCTGAC
ATGGTGAAGAAGGTTCTGCTCGGTAGCTTGACCTCGTACGTTGAAGCTCCTTGG
